# Proceedings Chicago Academy Medical Sciences

**Published:** 1860-05

**Authors:** Wm. Scott Denniston

**Affiliations:** Assistant Secretary


### CHICAGO ACADEMY OF MEDICAL SCIENCES.

Regular meeting, April 6th, (Friday) 1860.

The minutes of the last meeting were read and approved.

Dr. C. G. Smith, presented a specimen of Schirrus of the

Pylorus for the inspection of the Fellows present. He was unable to give any history of the case.

Dr. V. L. Hurlbut read a paper, urging upon the Fellows of the Academy the propriety of giving a fair trial to the "active principles of medicinal plants, as prepared by B. Keith & Co.," stating that he had used them with success.

Dr. Bevan inquired if among the active principles there was any article which could be substituted for the preparation of mercury, especially in the treatment of veneral diseases.

Dr. Hurlbut replied that there were articles which produced the effects of mercurial preparations, instancing Podophyllin, Leptandrin, and a few others. In the treatment of veneral diseases, he used the Stillingia and Phytolacca.

Dr. Graham said that he had used these remedies to some extent, but that in his hands they had not accomplished what was promised for them. Podophyllin when used in the doses laid down in Keith's manuel, almost always produced unpleasant nausea and griping.

Dr. Hurlbut said that he had met with no unpleasant effects from the use of Podophyllin; he rarely gave more than a quarter of a grain at a dose, unless he wished for a decided cathartic effect, and usually combined it with the Asclepin. Another remedy which he considered very valuable was the Gelseminum, in doses of not more than half a grain; he considered it preferable to the preparations of opium, in that it left no unpleasant effect. Before using any of these active principles, it was necessary to neutralize any acidity of the stomach that might be present.

Dr. Blake had seen a paper by Prof. Kirtland, in the *Cleveland Medical Journal*, in which he stated that he had used Podophyllin and Leptandrin almost to the exclusion of Mercury. Dr. B. had used them successfully himself.

Dr. Wickersham thought that Prof. Kirtland used them only in cases of torpidity of the Liver.

With reference to Prof. Kirtland's paper, Dr. Ingalls stated that the Ohio Medical Society, discrediting, had refused to publish it.

On motion, the paper of Dr. Hurlbut was referred to the Committee on Materia Medica.

Dr. Hamill read a paper (a continuation of one presented at the last meeting) on Sulphate of Quinine and some of its effects.

Dr. Holmes inquired whether any attempt had ever been made to ascertain the amount of moisture thrown off by the human lungs.

Dr. Hamill thought not.

Dr. Bloodgood had read somewhere that the amount of moisture thrown off by insensible perspiration was five pounds in twenty-four hours.

Dr. Ingalls thought it was not safe to assume that there was no moisture thrown off by the skin during fever, because the skin was hot and dry; he thought that there was insensible perspiration.

Dr. Jones asked if Dr. Hamill ever considered Quinine a sudorific.

Dr. Hamill thought that the perspiration usually following its administration was not an effect of the quinine.

Dr. Bevan had never seen any legitimate tonic effect follow the use of Quinine; he had never known it to have any effect on night sweats even; he considered its effect wholly anti-periodic, and agreed fully with the views of Dr. Hamill.

Dr. Freer expressed his concurrence with the views advanced by Dr. Hamill, as far as related to the practical application of the remedy.

On motion, the paper of Dr. Hamill was referred to the Committee on Practical Medicine.

Dr. Bevan reported a case of Diphtheria; he thought the disease should be treated with general and local anti-phlogistics, notwithstanding the high authorities for an opposite course.

Dr. Byford had seen three cases of diphtheria during the year; he thought that in the treatment of this and similar inflammations, too much stress had been laid on the use of tonics; that it was better to begin the treatment with antiphlogistics, and end by supporting the patient, if necessary. He considered general treatment more important than local applications.

Dr. Freer had seen one case almost identical with that reported by Dr. Bevan. His treatment was the local application of Chlorate of Potash and Muriatic Acid every two or three hours, and the internal use of Chlorate of Potash slightly acidulated with Muriatic Acid. The child improved under the treatment, but relapsed in a few days and sunk, with passive hœmorrhage from the bowels. He inquired if passive hœmorrhage was usual in these cases.

Dr. Graham had seen passive hœmorrhage from the nose in two fatal cases. He thought that an antiphlogistic treatment was sometimes necessary in this disease.

Dr. Bevan next reported a fatal case of spasm of the glottis. The only symptoms during life were an occasional interruption of respiration, with slight crowing sound.

Dr. Byford had attended a case a year ago, similar to that reported by Dr. Bevan, except that the crowing respiration was absent.

Dr. C. G. Smith had attended a somewhat similar case, which resulted in laryngismus stridulus. The child after presenting all the symptoms of asphyxia would entirely recover, and continue well for some days, when the spasms would recur. All the anti-spasmodics were used without success. The case passed out of his hands, and he learned afterwards, died in one of the spasms.

Dr. Bevan considered the anti-spasmodics utterly useless in these cases of eclampsic disease. He never found them of any benefit, except where a hysteric tendency was manifested.

The two reports of Dr. Bevan were referred to the Committee on Pathology.

Dr. C. G. Smith read a letter from Dr. Geo. D. Wilbur, of Mineral Point, Wisconsin, which accompanied a package containing a human stomach and liver, and in which the writer requested a report of a microscopical examination of the specimens.

On motion the specimens were referred to the Committee. The Corresponding Secretary was instructed to inform Dr. Wilbur that they had been so disposed of, and to request a history of the case from which they had been taken.

On motion, the President appointed Drs. Rauch, C. G. Smith, Blake, and Bevan, delegates to the State Medical

Society. Drs. Johnson, Ingalls and Rauch, were elected delegates to the National Convention for the revision of the Pharmacopeia.

On motion, adjourned to meet on Friday evening, April 20.

(Signed) WM. SCOTT DENNISTON,
*Assistant Secretary.*

